# Adherence to Established Treatment Guidelines Among Unguided Digital Interventions for Depression: Quality Evaluation of 28 Web-Based Programs and Mobile Apps

**DOI:** 10.2196/16136

**Published:** 2020-07-13

**Authors:** Stefan Bubolz, Gwendolyn Mayer, Nadine Gronewold, Thomas Hilbel, Jobst-Hendrik Schultz

**Affiliations:** 1 Department of General Internal Medicine and Psychosomatics Heidelberg University Hospital Heidelberg Germany; 2 Westphalian University of Applied Sciences Gelsenkirchen Germany

**Keywords:** web-based interventions, depression, mHealth, mental health, telemedicine, mobile phone, eHealth, electronic mental health, online therapy

## Abstract

**Background:**

Web-based interventions for depression have been widely tested for usability and functioning. However, the few studies that have addressed the therapeutic quality of these interventions have mainly focused on general aspects without consideration of specific quality factors related to particular treatment components. Clinicians and scientists are calling for standardized assessment criteria for web-based interventions to enable effective and trustworthy patient care. Therefore, an extensive evaluation of web-based interventions at the level of individual treatment components based on therapeutic guidelines and manuals is needed.

**Objective:**

The objective of this study was to evaluate the quality of unguided web-based interventions for depression at the level of individual treatment components based on their adherence to current gold-standard treatment guidelines and manuals.

**Methods:**

A comprehensive online search of popular app stores and search engines in January 2018 revealed 11 desktop programs and 17 smartphone apps that met the inclusion criteria. Programs and apps were included if they were available for German users, interactive, unguided, and targeted toward depression. All programs and apps were tested by three independent researchers following a standardized procedure with a predefined symptom trajectory. During the testing, all web-based interventions were rated with a standardized list of criteria based on treatment guidelines and manuals for depression.

**Results:**

Overall interrater reliability for all raters was substantial with an intraclass correlation coefficient of 0.73 and Gwet AC1 value of 0.80. The main features of web-based interventions included mood tracking (24/28, 86%), psychoeducation (21/28, 75%), cognitive restructuring (21/28, 75%), crisis management (20/28, 71%), behavioral activation (19/29, 68%), and relaxation training (18/28, 64%). Overall, therapeutic meaningfulness was rated higher for desktop programs (mean 4.13, SD 1.17) than for smartphone apps (mean 2.92, SD 1.46).

**Conclusions:**

Although many exercises from manuals are included in web-based interventions, the necessary therapeutic depth of the interventions is often not reached, and risk management is frequently lacking. There is a need for further research targeting general principles for the development and evaluation of therapeutically sound web-based interventions for depression.

## Introduction

### Background

Major depression is the leading cause of disability and affects more than 300 million people worldwide [1], with a lifetime prevalence up to 20.6% for different populations [2-4]. Even minor forms of depression are associated with reduced quality of life [5], increased mortality [6], and functional impairments [7]. Additionally, the economic consequences of depression are extensive, and the treatment costs are increasing [8]. Although psychotherapy is an established, evidence-based treatment for depression [9-12], many individuals in need of mental health services do not receive adequate treatment [13,14]. Access barriers to treatment that are often associated with mental illness include limited availability of psychotherapists, long waiting lists, high costs of treatment, and fear of stigmatization [15-19]. Web-based interventions are considered a promising model to address this treatment gap [13,14]. They provide immediate support at any time and place, are cost-effective, and can easily be administered to a vast majority of people, thus reducing barriers and enabling high accessibility to treatment [20-22].

The number of concepts for web-based interventions has been gradually increasing, including telemedicine, electronic health apps, smartphone health apps, or mobile health apps. Furthermore, web-based interventions can be classified according to various criteria, ranging from smartphone apps for mere self-management to guided programs that include the support of a therapist through either asynchronous communication (such as email) or synchronous communication (such as videoconferencing). In many cases, a clear and distinctive assignment of a web-based intervention to a single type is not possible [23]. In this study, we followed the definition of a web-based intervention as an intervention program that is (1) available via the internet, (2) provides interactive components, (3) comprises health-related material, (4) aims to create positive change, and (5) aims to improve knowledge and understanding of a disease [24]. Following this definition, we included interventions that provide initial access via the internet, whereas it was not mandatory for the intervention to remain connected to the internet throughout its course. Additionally, we distinguished between smartphone apps and desktop programs because of their differences in technological features (eg, use of push notifications in smartphones and better readability of text on desktop screens).

The increase of innovative technologies in the field of web-based interventions shows great promise for potential in improving quality of life for people suffering from depression. Web-based treatment for depression has proven to be effective in reducing symptoms [25-29], reducing stigma [30-32], and improving depression literacy [31,32]. Furthermore, web-based interventions can deliver therapy to users independent of location with 24-hour accessibility [33].

Consumers searching for mental health apps most likely use social media, online search engines, or web forums [34]. A recent study identified more than 380,000 health apps worldwide, 28% of which are targeted toward mental health and behavioral disorders [35]. For users in the United States, more than 500 apps for depression are currently available [36]. Nevertheless, only a small proportion of the web-based interventions available for depression are supported by evidence-based studies [37], and research on web-based interventions has thus far focused mainly on validating single products [38]. Criticisms of web-based interventions include poor methodological quality of effectiveness studies such as by comparing interventions with wait-list control groups or by showing evidence only through analysis of short-term effects [23,39].

When web-based interventions lack evidence or disregard standards and guidelines for psychotherapeutic treatment, they may offer ineffective care or cause direct harm to users, such as by providing adverse advice or diverting users from accessing effective treatment [40-42]. Another problem that many web-based interventions face is a high attrition rate because many users drop out after a few days or weeks of using the intervention [43,44]. Reasons for low user engagement, especially with smartphone-based mental health apps, were recently summarized in a review by Torous et al [45]. Low usability, lack of user-centric design, concerns about privacy, lack of trust by users and by clinicians, and concerns about unhelpfulness in emergencies were the most relevant issues identified. Thus, “building trust through standards” [45] is considered to be the most important, yet challenging, goal for developers of high-quality web-based interventions.

Clinicians and scientists are calling for standardized assessment criteria for web-based interventions to enable effective and trustworthy patient care [23,46]. As a result, professional associations such as the Anxiety and Depression Association of America have started to develop their own criteria [47], but further research is necessary.

### Previous Research on Quality Measures for Web-Based Interventions

There are several approaches adopted in previous research to develop appropriate assessment tools for ensuring the therapeutic quality of web-based interventions for depression.

Renton et al [48] provided a scoping review and evaluated web-based interventions for depression on a 28-point rating system, covering aspects such as usability, accessibility, and type of tools used (eg, worksheets and assessments). They found high variability among the web-based interventions, and that most of these interventions used cognitive behavioral therapy (CBT) as the therapeutic approach with mood assessments and homework sheets implemented as the primary interactive tools. These results are supported by another recent review showing that 85% of the web-based interventions for depression implemented CBT techniques [49].

One prominent and often used scale is the Mobile App Rating Scale (MARS) [50], a multidimensional measure that includes 19 items on 4 objective quality indicators for apps: engagement, functionality, esthetics, and information quality. Additionally, 4 items measure the subjective quality of an app [50]. MARS has been validated with well-being apps and the instrument showed high internal consistency (Cronbach α=.90) [50]. The MARS has been used for a broad scope of apps, including smoking cessation apps [51], fitness apps for cancer patients [52], and German depression apps [53].

However, Baumel et al [54] noted that current criteria-based rating scales such as MARS lack an assessment of therapeutic alliance principles. Therefore, the authors developed a detailed evaluation tool for mobile and web-based health interventions termed “Enlight,” which includes a quality assessment section with 6 constructs: usability, visual design, user engagement, content, therapeutic persuasiveness, and therapeutic alliance (ie, basic acceptance and support). The instrument was validated with mobile apps and web-based programs for behavioral change in the case of medical illness or mental health, and showed high internal consistency (Cronbach α=.83 to .90). Although Enlight recognizes the therapeutic content of web-based interventions, only 4 items are dedicated to this issue.

Previous studies have measured the therapeutic quality of web-based interventions only at a general level, reflecting the overall impression of raters, without specific analysis of particular treatment components (eg, behavioral activation, cognitive restructuring) or therapeutic approaches. Although the majority of web-based interventions implement CBT as the therapeutic approach [48,49], we found only one study that examined the realization of a specific component of CBT in web-based interventions in detail (ie, behavioral activation) [55]. The authors found that the utility of these interventions is questionable, because only a few adhere to the core principles of CBT.

Qu et al [56] systematically examined the functionalities of depression apps and found that 31% of the apps evaluated offer depression screening, 66% offer tracking functionalities (eg, mood tracking), and 83% offer some form of therapeutic intervention (eg, psychoeducation or thought diaries). However, the therapeutic quality of these interventions was not the focus of their study.

To our knowledge, no studies published to date have aimed at specifically evaluating the therapeutic quality of web-based interventions at the level of several individual treatment components. As clinicians and scientists are calling for standardized assessment criteria, and previous measures did not examine the individual treatment components of web-based interventions for depression at an in-depth level, in the present study, we evaluated the therapeutic quality of currently available web-based interventions for depression.

### Study Aim

The aim of this study was to evaluate the quality of unguided web-based interventions for depression at the level of individual treatment components based on their adherence to gold-standard treatment guidelines and manuals. Our primary research question was: How extensively do web-based interventions for depression adhere to established treatment standards? This includes (1) which core treatment components of established guidelines were realized in the web-based intervention, (2) how close did the treatment components follow the recommendations of the guidelines regarding their delivery mode and instruction, and (3) how potential risks in the treatment process are managed. We did not aim to provide recommendations for or against individual programs or apps. As a means to achieve the study aims, we examined current treatment guidelines and manuals for depression, and developed a questionnaire that comprises standardized testing criteria.

## Methods

### Search Strategy and Inclusion Criteria

In January 2018, three researchers independently searched for online treatment programs targeted at depression. Since we aimed at searching for web-based interventions from a patient perspective, the searches were carried out in three major app stores (Google Play, iTunes, Windows Store) and four broadly popular search engines (Google, Yahoo, Bing, Duckduckgo). Additionally, we investigated the Beacon website [57] (an Australian platform for health apps) and HealthOn [58] (a German platform for health apps) for web-based interventions meeting our inclusion criteria. We did not search published evidence in the scientific literature because it is not clear when a web-based intervention reported in the literature becomes available on the market. Further, we excluded web-based interventions that required participation in a study. We found that it is more likely for patients to participate in a noncommittal web-based intervention with easy access when they are looking for online treatment options, which is not the case in a clinical study. The search terms used were “depression” or the wildcard search term “depress*” either by itself or in combination with one or more of the following terms: “online,” “web-based,” “treatment,” and “program.”

Apps or desktop programs were included in our analysis based on the following criteria: (1) claimed to provide treatment or support for depression, (2) were accessible to the public via the internet (with or without fee), (3) had an interactive component (ie, were not purely educational) and required user participation or input (eg, homework, worksheets, mood assessments), (4) were available in English or German (because these were the fluently spoken languages by all raters), (5) provided a scientific basis for their treatment (eg, based on CBT), and (6) were targeted to adults (older than 18 years). We adapted inclusion criteria that can be found in previous scoping reviews on web-based interventions for depression in the scientific literature [48] to fit the purpose of this study (criteria 1-4), and added further criteria that we considered necessary to fulfill the particular objective of this study (criteria 5-6).

Apps or desktop programs were excluded from this study if they (1) only provided information regarding depression (ie, psychoeducation) and offered no further intervention, (2) did not claim to be based on a scientific background, (3) did not specifically target depression (ie, were targeted at other or multiple disorders), (4) were not accessible to the public (ie, programs for patients of a specific clinic), (5) targeted health care professionals for training purposes, (6) offered only mood tracking, (7) were only available for research purposes (ie, user must be enrolled in a study to access the program), (8) offered no treatment program, (9) could not be completed within the home or private setting (ie, must attend classes), (10) required the participant to get in touch with a counselor (ie, webcam counseling, therapy sessions, chatroom counseling), and (11) refused participation in this study.

Programs were screened for relevance based on the title, description, and available further information given on the respective webpages (eg, frequently asked questions, videos, or screenshots).

In line with previous work that identified web-based intervention programs for depression [48], we ceased the search when no new programs could be identified within five consecutive pages of search results.

### Testing Criteria

The original questionnaire used in this study was developed in German. A translated version of the complete questionnaire can be found in Multimedia Appendix 1.

To obtain objective testing criteria, we examined current guidelines and manuals that are established in the treatment of depression, including the S3 and National Health Care guideline on unipolar depression [59,60], Beck’s manual for CBT [61], and the “Coping with Depression” course [62].

The treatment guidelines were examined by three researchers who each extracted treatment components and developed a set of objectively ratable criteria that could possibly be implemented by a web-based intervention. Subsequently, the researchers compared their criteria and solved discrepancies by consensus. The criteria were then reviewed by medical experts of the psychosomatic and psychosocial field, who provided comments and compared the criteria to the guidelines once more. The researchers used this information to revise the criteria conclusively, and any discrepancies were solved by consensus.

Following this process, we chose CBT components as superordinate categories in our questionnaire because CBT was identified as the most frequent approach in web-based interventions for depression [48,49]. Based on the guidelines and manuals, we expected the following components to occur in web-based interventions that claim to be evidence-based: behavioral activation, cognitive restructuring, psychoeducation, mood tracking, journal keeping, relaxation training, social skills training, resource activation, and crisis management.

The questionnaire includes a general and a specific part for each component, except for resource activation and crisis management. The general part comprises the same items for each component and aims to address basic principles such as transparency, understandability, or therapeutic potential. Example items for the general part are: “The therapeutic background for the intervention is presented,” “The instruction can easily be understood,” and “Possible difficulties regarding the intervention are addressed.”

The specific part comprises items unique to the therapeutic tools or the theoretical rationale of each component. For example, in case of cognitive restructuring, items for the specific part were: “The principle of automatic thoughts is explained clearly,” “The program suggests alternative thoughts for negative thoughts,“ and “The program offers the option to create a daily record of negative thoughts.”

The questionnaire closes with crisis management and resource activation. For crisis management, items concerning the instruction, therapeutic background, digital implementation, and therapeutic meaningfulness were included from the general part. Further items asked for emergency contacts, relapse prevention, contact to support groups, and behavior in case of suicidality. Resource activation comprises 4 items concerning the identification of resources and their reflection in the course of the intervention.

The developed criteria do not claim to be exhaustive. Rather, we tried to find as many objectively ratable aspects as possible that can characterize a good and CBT-conforming psychotherapy. The absence of a treatment component or a particular criterion does not necessarily imply poor quality of the intervention. However, the presence of many criteria can indicate high quality.

### Testing Procedure

The three independent researchers who conducted the search also rated the included web-based interventions following a standardized testing procedure. Raters followed the recommendations for usage given by each web-based intervention regarding the time and duration of usage (eg, daily usage for several minutes or weekly completion of session). When programs consisted of sessions, the programs were tested until all sessions relevant to the identified treatment components were completed. When provided, we followed the suggested order and instructions of each web-based intervention (eg, completing 12 sessions over 12 weeks). When a web-based intervention was designed to be used at will and no clear point of completion of the treatment could be identified, it was tested in an intuitive manner (ie, interventions were completed in the order in which they appeared to the user) until each intervention was completed at least once.

When symptom questionnaires, mood tracking, or progress tracking were included in a web-based intervention, raters responded with a set of previously defined symptoms. At the beginning of the web-based intervention, raters responded with a moderate symptom severity and changed the occurrence and severity of symptoms during the course of the intervention. Symptoms were chosen following a moderate depression score according to Beck’s depression inventory II [63]. When repeated assessments were included during the progression of a web-based intervention, each researcher submitted a deterioration of symptoms, characterized by increased severity of core symptoms and the addition of suicidal thoughts, to test how the web-based intervention responded to suicidal tendencies and how it provided risk management. At later stages, each rater submitted an improvement of symptoms, characterized by mild degrees of core symptoms and loss of additional symptoms, to test how the program responded to treatment progress.

All raters have a degree in psychology and backgrounds in psychotherapy (CBT, psychodynamic therapy, and systemic therapy). Web-based interventions were randomly allocated to the raters and each intervention was independently tested by two raters.

No ethical approval was required because no human participants outside the researchers took part in this study.

### Statistical Analysis

Descriptive statistical analysis (including means, SDs, and frequencies) and intraclass correlation coefficient (ICC) estimates were calculated using SPSS statistical package version 24 (IBM Corp Released 2016, IBM SPSS Statistics for Windows, Armonk, NY, USA).

To assess the interrater reliability for all reported metric items, ICC estimates and their 95% CIs were calculated for each rater pair based on a one-way random-effects model with absolute agreement.

To assess the interrater reliability for all reported dichotomous items, Gwet AC1 statistic was calculated using formula 4.1 of Gwet [64] through the WINPEPI software developed by Abramson [65]. Gwet AC1 statistic is a chance-corrected measure of the extent of agreement between raters but has been recommended for use because it is less influenced by differences in individual rater tendencies to give positive ratings and by differences in the prevalence of the response categories, thus making it more robust and less biased compared to other metrics [66-68].

## Results

### Search Results

**Figure 1:**
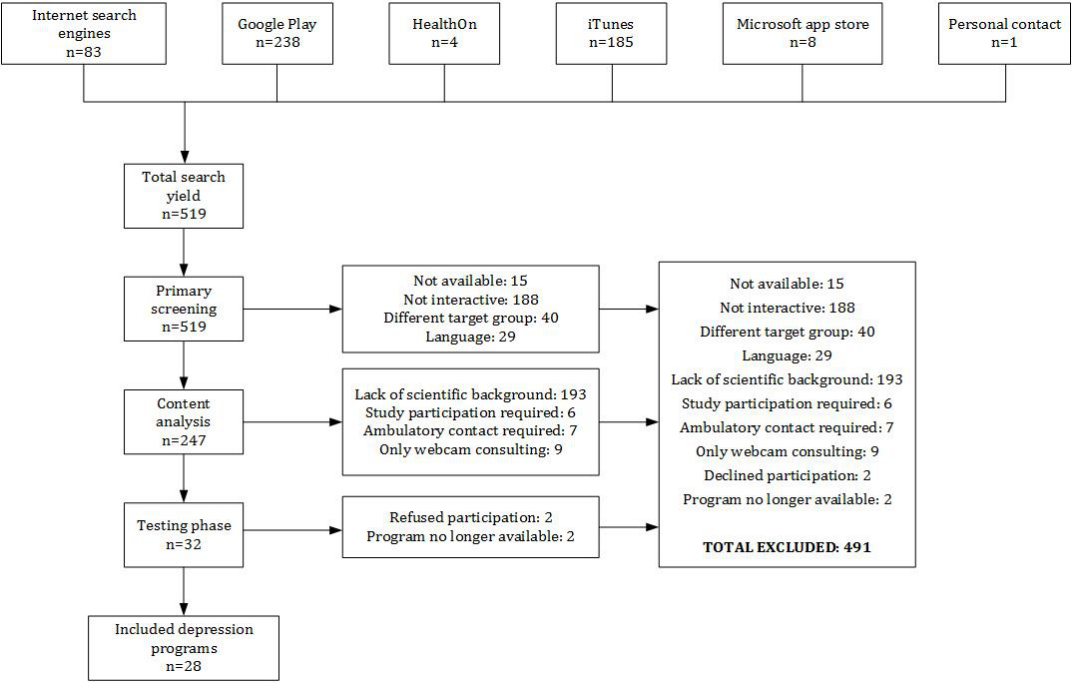
Flowchart of the screening process to identify desktop programs and apps.

The initial search yielded 519 web-based interventions. Thirty-two of these were identified to meet our inclusion criteria. Discrepancies regarding the identified programs were resolved by consensus. During our testing period, 2 programs withdrew their participation from the study and 2 programs were no longer publicly available, leaving 28 programs that were ultimately tested (see Figure 1 for a flowchart of the screening and inclusion process). The 28 programs included 11 desktop programs and 17 smartphone apps. A list of the tested web-based interventions can be found in Multimedia Appendix 2.

### Interrater Reliability

Mean ICC estimates and Gwet AC1 statistics for all reported metric items are shown in Table 1. The overall range for ICC estimates was 0.66 to 0.79. According to the guideline developed by Koo and Li [69], mean ICC estimates in this study were indicative of moderate to good interrater reliability for all rating pairs. The overall range for the Gwet AC1 statistic was 0.75 to 0.84. Concordance for Gwet AC1 statistic is measured on the same scale as used for Landis and Koch’s κ criteria [70,71]; therefore, interrater reliability for the dichotomous items in this study can be considered to be substantial.

**Table 1:** Intraclass correlation (ICC) estimates and Gwet AC1 statistics for all rating pairs.

Rating pair	ICC (95% CI)	Gwet AC1 (95% CI)
Rating pair 1 and 2	0.79 (0.72-0.87)	0.84 (0.79-0.89)
Rating pair 1 and 3	0.70 (0.58-0.83)	0.75 (0.68-0.83)
Rating pair 2 and 3	0.66 (0.51-0.81)	0.80 (0.74-0.87)
Overall	0.73 (0.67-0.79)	0.80 (0.76-0.84)

### General Results

An overview of the identified treatment components for each web-based intervention can be found in Multimedia Appendix 3. Table 2 depicts the relative number of treatment components for each web-based intervention. We did not include resource activation in this summary because no web-based intervention assigned a specific module to resource activation, but rather implemented resource activation in several treatment components.

**Table 2:** Absolute numbers and respective availability of treatment components for desktop programs (N=11) and apps (N=17).

Treatment component	Desktop programs, n (%)	Apps, n (%)
Behavioral activation	9 (82)	10 (59)
Cognitive restructuring	8 (73)	13 (76)
Psychoeducation	10 (91)	11 (65)
Mood tracking	11 (100)	13 (76)
Journal keeping	4 (36)	8 (47)
Relaxation training	8 (73)	10 (59)
Social skills training	6 (55)	2 (12)
Crisis management	9 (82)	11 (65)

Taken together, the presentation of the therapeutic background of interventions received higher ratings in desktop programs (mean 4.13, SD 1.17) than in apps (mean 2.92, SD 1.46) and moderate ratings overall (mean 3.47, SD 1.46). Regarding the understandability of instructions, desktop programs received higher ratings than apps, and high ratings were given desktop programs and apps overall (mean 4.18, SD 1.15). The digital implementation of interventions was rated as moderate for both desktop programs and apps (mean 2.90, SD 1.17). Overall, interventions received moderate ratings for their therapeutic meaningfulness for desktop programs and apps (mean 3.54, SD 1.15). However, discrepancies regarding the therapeutic quality were identified between single interventions, with desktop programs receiving higher scores for behavioral activation, cognitive restructuring, and psychoeducation than apps, and similar scores for mood tracking and relaxation training (see Table 3 and Table 4 for detailed results).

**Table 3:** Mean (SD) scores of assessments of the therapeutic quality in interventions of desktop programs.

Item	Overall^a^	BA^b^	CR^c^	PE^d^	MT^e^	JK^f^	RT^g^	SST^h^	CM^i^
“The therapeutic background for the intervention is presented.”	4.13 (1.17)	4.89 (0.32)	4.94 (0.25)	3.65 (1.40)	3.36 (1.40)	4.25 (0.71)	4.13 (1.15)	4.25 (0.87)	4.00 (1.19)
“The instruction can easily be understood.”	4.70 (0.62)	5.00 (0.00)	4.81 (0.40)	4.70 (0.57)	4.59 (0.85)	4.63 (0.74)	4.56 (0.73)	4.58 (0.67)	4.67 (0.59)
“The (digital) implementation of the intervention is adequate.”	3.04 (1.09)	3.28 (0.83)	3.63 (1.03)	3.20 (1.11)	2.55 (1.06)	3.00 (1.31)	2.56 (1.03)	3.25 (1.14)	3.11 (0.83)
“The intervention is therapeutically meaningful.”	3.92 (1.07)	4.56 (0.62)	4.69 (0.60)	4.05 (1.05)	3.14 (1.04)	4.00 (0.93)	3.31 (1.08)	3.83 (1.47)	3.94 (0.73)

^a^Overall: includes all desktop programs.

^b^BA: behavioral activation.

^c^CR: cognitive restructuring.

^d^PE: psychoeducation.

^e^MT: mood tracking.

^f^JK: journal keeping.

^g^RT: relaxation training.

^h^SST: social skills training.

^i^CM: crisis management.

**Table 4:** Mean (SD) scores on assessments of the therapeutic quality in interventions of smartphone apps.

Item	Overall^a^	BA^b^	CR^c^	PE^d^	MT^e^	JK^f^	RT^g^	SST^h^	CM^i^
“The therapeutic background for the intervention is presented.”	2.92 (1.46)	3.20 (1.32)	3.42 (1.42)	2.68 (1.59)	2.58 (1.45)	3.19 (1.33)	2.80 (1.47)	3.00 (2.31)	2.59 (1.40)
“The instruction can easily be understood.”	3.74 (1.31)	3.80 (1.15)	3.77 (1.28)	3.64 (1.47)	4.04 (1.08)	4.06 (1.12)	3.80 (1.40)	3.50 (1.91)	3.18 (1.50)
“The (digital) implementation of the intervention is adequate.”	2.83 (1.28)	2.80 (1.47)	2.73 (1.25)	2.68 (1.52)	3.04 (1.11)	3.19 (1.05)	2.75 (1.29)	2.00 (0.82)	2.36 (1.00)
“The intervention is therapeutically meaningful.”	3.22 (1.12)	3.50 (1.15)	3.38 (1.17)	3.36 (1.26)	3.08 (1.06)	3.25 (0.78)	3.05 (1.05)	3.75 (0.96)	2.86 (1.28)

^a^Overall: includes all apps.

^b^BA: behavioral activation.

^c^CR: cognitive restructuring.

^d^PE: psychoeducation.

^e^MT: mood tracking.

^f^JK: journal keeping.

^g^RT: relaxation training.

^h^SST: social skills training.

^i^CM: crisis management.

### Behavioral Activation

Interventions aimed at behavioral activation were included in 9 desktop programs and 10 apps. All behavioral activation interventions suggested activities, including activating activities and relaxing activities, whereas fewer interventions suggested social activities (16/19, 84%).

The suggested activities were rated to be easy to realize (mean 4.47, SD 0.89) and pleasant (mean 4.26, SD 0.89). Complex activities were rated as not divided into achievable intermediate steps (mean 1.74, SD 1.33). Difficulties that can occur in the realization of suggested activities were rated as not sufficiently addressed (mean 2.24, SD 1.52).

The relative share of web-based interventions realizing further features of behavioral activation can be found in Table 5.

**Table 5:** Absolute numbers and relative share of web-based interventions addressing specific features of behavioral activation in desktop programs (N=9) and apps (N=10).

Features of behavioral activation	Desktop programs, n (%)	Apps, n (%)
Difficulty levels for activities	1 (11)	1 (10)
Push notifications	9 (100)	2 (20)
Add individual activities	9 (100)	9 (90)
Schedule activities	9 (100)	5 (50)
Reminders for activities	9 (100)	4 (40)
Check completed activities	1 (11)	1 (10)
Reference to past activities	2 (22)	3 (30)

### Cognitive Restructuring

Cognitive restructuring was included in 8 desktop programs and 13 apps. The explanation of the principle of automatic thoughts was rated to be more clear in desktop programs (mean 4.44, SD 1.15) than in apps (mean 2.92, SD 1.57). Beck’s cognitive triad was addressed in desktop programs (mean 3.00, SD 1.41), but less often in apps (mean 1.50, SD 1.14).

Desktop programs and apps presented typical negative thoughts (8/8, 100% and 10/13, 77%, respectively), suggested alternative thoughts (8/8, 100% and 8/13, 62%, respectively), and suggested that the user write down individual negative thoughts (8/8, 100% and 13/13, 100%, respectively) as well as alternative thoughts (8/8, 100% and 10/13, 77%, respectively).

All cognitive restructuring interventions offered the option of keeping a daily thought protocol (21/21, 100%), which included the situation (19/21, 90%), feelings (17/21, 81%), negative thoughts (19/21, 90%), alternative positive thoughts (15/21, 71%), and the result of positive thoughts (16/21, 76%).

Cognitive distortions were addressed in both desktop programs and apps. An overview of the relative share of web-based interventions addressing cognitive distortions can be found in Table 6.

**Table 6:** Absolute numbers and relative share of web-based interventions addressing cognitive distortions for desktop programs (N=8) and apps (N=13).

Type of cognitive distortion	Desktop programs, n (%)	Apps, n (%)
Should statements	7 (88)	10 (77)
Focus on the negative	7 (88)	10 (77)
Disqualifying the positive	7 (88)	8 (62)
Personalization	3 (38)	11 (85)
Labeling and mislabeling	6 (75)	9 (69)
Catastrophizing	7 (88)	9 (69)
Magnification and minimization	6 (75)	8 (62)
Emotional reasoning	6 (75)	10 (77)
Jumping to conclusions	7 (88)	11 (85)
Polarized thinking	7 (88)	10 (77)
Overgeneralization	8 (100)	10 (77)

### Psychoeducation

Psychoeducation was implemented in 10 desktop programs and 11 apps, which included explanations of depressive symptoms in both desktop programs (mean 3.90, SD 1.29) and apps (mean 3.35, SD 1.53). An explanatory model for the development of depression was provided in desktop programs (mean 3.95, SD 1.40) and apps (mean 3.48, SD 1.59). Low ratings were found for whether the user received support in the development of an individual explanatory model for desktop programs (mean 1.80, SD 1.24) and apps (mean 1.22, SD 0.52).

Both desktop programs and apps conveyed that depression is well treatable (mean 4.25, SD 1.12 and mean 3.30, SD 1.40, respectively) and that the user can overcome depression (mean 4.60, SD 0.75 and mean 3.30, SD 1.40, respectively).

Further evaluations regarding psychoeducation can be found in Table 7.

**Table 7:** Mean (SD) scores for evaluation of psychoeducation features in web-based interventions.

Item	Overall	Desktop programs	Apps
“The program appropriately conveys that there may be fluctuations in the course of treatment.”	2.45 (1.35)	2.89 (1.49)	2.09 (1.13)
“The possible relationship between depression and anxiety is adequately conveyed.”	2.16 (1.29)	2.45 (1.36)	1.91 (1.20)
“The role of avoidance behavior is explained clearly.”	2.62 (1.58)	3.21 (1.40)	2.65 (1.58)
“The role of social isolation is explained clearly.”	2.91 (1.44)	3.20 (1.24)	2.65 (1.58)
“The possibly increased perception of physical symptoms associated with depression is explained clearly.	1.84 (1.31)	1.75 (1.16)	1.91 (1.44)
“Possible somatic causes of physical symptoms are adequately addressed.”	1.91 (1.49)	1.85 (1.63)	1.96 (1.40)

### Mood Tracking

Mood tracking was implemented in 24 web-based interventions, including 11 desktop programs and 13 apps. When a web-based intervention reminded the user to report his or her mood on a regular basis, the reminders occurred less than every 6 hours in most cases (19/24, 79%). The queries of the mood were visualized in desktop programs (mean 3.41, SD 1.37) and apps (mean 3.85, SD 1.32), and took into account common diagnostic criteria for depression (desktop programs mean 3.45, SD 1.50; apps mean 2.50, SD 1.56). The mood queries fulfilled our criteria for scaling (ie, offering at least 5 options) in some desktop programs (8/11, 73%) and apps (11/13, 85%).

Low ratings were given for the explanation of why it can be important to track small periods of time (to detect triggers for specific moods; overall mean 1.27, SD 0.79) or large periods of time (to detect trends in the mood progression; overall mean 1.08, SD 0.35). When mood progression was visualized (eg, as a curve), small periods of time (eg, the last few hours) could be chosen in 10 web-based interventions, including 3/11 (27%) of desktop programs and 7/13 (54%) of apps, and longer periods of time (eg, more than 1 week) could be chosen in 12 web-based interventions, including 5/11 (45%) desktop programs and 7/13 (54%) apps. When mood improvements were given, 4 web-based interventions highlighted improvements (3/11, 27% of desktop programs and 1/13, 8% of apps) and 8 web-based interventions suggested possible relations between mood and current events (2/11, 18% of desktop programs and 6/13, 46% of apps).

Overall, 11 web-based interventions asked the user about anxiety symptoms (6/11, 55% of desktop programs and 5/13, 38% of apps) and 15 web-based interventions asked the user about physical ailments (8/11, 73% of desktop programs and 7/13, 54% of apps). When physical ailments were assessed, 7 web-based interventions advised the user to consult a physician (5/11, 45% of desktop programs and 2/13, 15% of apps).

### Journal Keeping

Interventions that provided journal keeping were included in 4 desktop programs and 8 apps. Overall, the web-based interventions received low ratings for their explanation of which components the journal entries might contain (mean 1.96, SD 1.43) or for their explanation of how it can be helpful to note the positive aspects of the day (mean 2.38, SD 1.70). In most cases, journal entries could be entered through blank text boxes, although some web-based interventions suggested preset phrasings for particular aspects of the journal (7/12, 58%). Some web-based interventions took up journal entries in the progression of the program (2/12, 17%) or provided feedback on the content of journal entries (2/12, 17%).

### Relaxation Training

Overall, relaxation trainings were realized in 8 desktop programs and 10 apps. In both types of web-based interventions, mindfulness was suggested as a relaxation technique and the concept of mindfulness was explained in desktop programs (mean 3.00, SD 1.63) and apps (mean 2.85, SD 1.63). Other relaxation techniques that were suggested in the web-based interventions are summarized in Table 8.

When mindfulness was discussed in an intervention, 12 web-based interventions suggested to accept perceptions without judging them (5/8, 63% of desktop programs and 7/10, 70% of apps). Overall, 12 web-based interventions suggested to distance oneself from thoughts (5/8, 63% of desktop programs and 7/10, 70% of apps) and 7 web-based interventions suggested to perform mindfulness exercises as part of a daily routine (2/8, 25% of desktop programs and 5/10, 50% of apps). Additionally, 14 web-based interventions offered audio- or video-based mindfulness exercises (6/8, 75% of desktop programs and 8/10, 80% of apps).

The explanation of typical stressors received low ratings in desktop programs (mean 2.00, SD 1.14) and apps (mean 1.45, SD 0.76). One desktop program and one app offered the user to add personal stressors. The explanation of possible risks in performing mindfulness exercises (eg, the occurrence of unpleasant feelings) received low ratings for desktop programs (mean 1.81, SD 1.17) and apps (mean 1.30, SD 0.57). Similar results were found for the explanation of possible difficulties in performing mindfulness exercises (eg, boredom or falling asleep) in desktop programs (mean 2.00, SD 1.10) and apps (mean 1.80, SD 1.15).

**Table 8:** Absolute numbers and relative share of relaxation techniques suggested in web-based interventions for desktop programs (N=8) and apps (N=10).

Relaxation technique	Desktop programs, n (%)	Apps, n (%)
Mindfulness	8 (100)	8 (80)
Progressive muscle relaxtion	7 (88)	5 (50)
Meditation	4 (50)	8 (80)
Guided imagery journeys	1 (13)	3 (30)
Imagination exercises	2 (25)	5 (50)

### Social Skills Training

Social skills training was included in 6 desktop programs and 2 apps. When social skills training was included, users were encouraged to perform a change of perspective in desktop programs (mean 4.25, SD 1.22) and apps (mean 4.50, SD 0.58).

When nonverbal or verbal components of social interaction were discussed (eg, maintaining eye contact or paying attention to voice modulation), desktop programs received higher ratings for their explanations (mean 3.08, SD 1.38 for nonverbal components and mean 3.42, SD 1.44 for verbal components) than apps (mean 1.50, SD 0.58 and mean 1.25, SD 0.50, respectively).

Six desktop programs and one app encouraged the user to perform exercises of social interaction (eg, paying somebody a compliment). The perception of social cues was rated higher in desktop programs (mean 2.92, SD 1.38) than in apps (mean 1.75, SD 0.96).

Both desktop programs and apps addressed the establishment and maintenance of social contacts (mean 3.17, SD 1.33 and mean 3.00, SD 1.42, respectively) and the assertion of one’s own wishes in social situations (mean 3.50, SD 1.62 and mean 3.25, SD 1.71, respectively).

### Crisis Management

Crisis management was included in 9 desktop programs and 11 apps. Nine desktop programs and 9 apps provided an emergency contact to the user (eg, phone numbers or contact details that are accessible at any time; 9/9, 100% and 9/11, 82%, respectively). One desktop program and one app required the user to provide an emergency contact in order to use the program (1/9, 11% and 1/11, 9%, respectively). To prevent relapses, 3/9 (33%) desktop programs and 3/11 (27%) apps suggested creating an individual list of warning signs. Contact to support groups was offered by 2/9 (22%) desktop programs and 3/11 (27%) apps.

When the mood in the mood tracking dropped immensely, 7/9 (78%) desktop programs and 4/11 (36%) apps suggested contacting the emergency contact. Three desktop programs and 5 apps suggested creating an emergency plan with individual measures (3/9, 33% and 5/11, 45%, respectively). When the mood dropped, 1/9 (11%) desktop program and 1/11 (9%) app suggested resorting to the emergency plan.

### Resource Activation

Although no web-based intervention evaluated in our study implemented a specific module for resource activation, this aspect was indirectly included within several other treatment components. A summary of evaluations regarding resource activation can be found in Table 9, including encouragement to identify individual resources, assistance in identifying resources, encouragement to reflect resources in their context, and uptake of resources in the progression of the web-based intervention. Among all web-based interventions included in this study, 3 desktop programs and 3 apps took up identified resources as the intervention progressed.

**Table 9:** Mean (SD) scores in the evaluation of resource activation features of web-based interventions.

Item	Overall	Desktop programs	Apps
“The user is encouraged to identify his/her own resources.”	2.76 (1.56)	3.45 (1.36)	2.30 (1.54)
“The program provides adequate assistance in identifying individual resources (eg, thinking about past successes).”	2.32 (1.60)	3.20 (1.51)	1.73 (1.39)
“The user is encouraged to reflect resources in their context (eg, origin story, typical situations, promoting or inhibiting factors).”	2.02 (1.39)	2.75 (1.41)	1.53 (1.17)

## Discussion

### Principal Results

The aim of this study was to evaluate the quality of unguided web-based interventions for depression at the level of individual treatment components based on their adherence to established treatment guidelines. We tested 28 web-based interventions with a self-developed, standardized list of criteria, which were based on gold-standard treatment guidelines and manuals for depression. Despite the high number of programs and apps for depression available through the commercial market that claim to follow a scientifically sound methodology, we found varying degrees of adherence to established treatment guidelines and manuals.

All web-based interventions included some of the interventions we expected in a CBT-based treatment, indicating conformance to treatment guidelines to some extent. As a result, our criteria were applicable, and we could gather insights about how the interventions were digitally realized. Overall, there is an evident trend for desktop programs to be rated as more adherent to treatment manuals than apps, which is in line with previous research. In a systematic review, Huguet et al [55] found that only 10% of depression apps seem to be consistent with evidence-based methods of CBT. However, their study focused on behavioral activation, which was only one part of our approach. In our study, cognitive restructuring, psychoeducation, and mood tracking were the most frequently realized components, which were included in more than 70% of the web-based interventions that we tested.

Our key findings reveal substantial variation regarding the therapeutic utility of these interventions, and interventions were diversely realized. For example, in treatment components targeting cognitive restructuring, the explanation of the principle of automatic thoughts was rated high in desktop programs, yet had a lower rating in apps. In contrast, Beck’s cognitive triad was addressed less often, although it can provide therapeutic background as an explanatory model for depression. Nevertheless, all cognitive restructuring interventions offered the option to keep a protocol of negative thoughts, as recommended in the manuals, and most of them included details such as the situation, feelings, negative thoughts, alternative positive thoughts, and result of positive thoughts.

When considering the use of mood tracking, we found that 79% of the web-based interventions that implemented mood tracking in our study inquired the mood only once every 6 hours or less frequently. Since retrospective recall of mood in people with depression is biased toward the negative [72,73], web-based interventions could be improved by adding more frequent mood inquiries. Additionally, frequent mood tracking throughout the day can help patients identify triggers for negative moods. However, it may be difficult for patients to track their mood frequently throughout the day on a desktop program. This could be complemented by using worksheets or combining desktop programs with app features. In particular, apps that have the opportunity to make ideal use of ecological momentary assessment could tap into their full potential by suggesting multiple mood inquiries throughout the day [74].

Out of all of the web-based interventions that we tested, 61% did not provide sufficient risk and crisis management when the mood dropped immensely. The most common reaction to a sudden mood drop was the recommendation to call emergency contacts or a crisis line. Yet, some web-based interventions did not react at all to severe mood drops. Although all of the web-based interventions self-identified as not suitable for suicidal patients, strong mood changes and suicidality are common symptoms in depression [75] that should not be completely excluded.

Another aspect that we noted is that the majority of web-based interventions asked the user about physical ailments, but not about anxiety symptoms. Web-based interventions should pay attention to and educate the user about anxiety symptoms and somatic diseases related to depression because there are high rates of comorbidity among depression, anxiety, and physical illness [76-78]. It would be beneficial if a web-based intervention asked the user about anxiety symptoms and provided information about treatment opportunities for anxiety so that users can reach out to professional help when they need it. Additionally, web-based interventions should recommend that users receive a proper somatic examination when somatic symptoms are present because some symptoms of depression can also be caused by physical illnesses (eg, lack of energy, difficulty in concentrating).

Some web-based interventions did not mention possible difficulties regarding the interventions they suggested. This can result in feelings of failure or disappointment when the user does not manage to complete the intervention. As an example, we found that 19 web-based interventions included behavioral activation and suggested the uptake of specific activities, but the raters evaluated that complex activities were not sufficiently divided into achievable intermediate steps, difficulties for the realization of suggested activities were not sufficiently addressed, and difficulty levels for the suggested activities were not sufficiently provided. Additionally, only 2 out of these 19 web-based interventions asked if an activity was completed. Altogether, this may result in the user feeling invalidated.

As some components of web-based interventions can be text-intensive and do not achieve proper risk management, ongoing engagement and motivation may be required for the user to follow the intervention, which may be very challenging for a depressed individual. Previous research found dissatisfaction and a lack of motivation to be a possible explanation when no benefits of a web-based intervention could be found [79]. Additionally, lack of a user-centric design and concerns about unhelpfulness in emergencies were identified as reasons for low user engagement in smartphone-based mental health apps [45].

Taken all together, we suggest that lack of adherence to treatment guidelines might be a reason why some users find no sufficient benefit in web-based interventions and discontinue the use after a short time, a phenomenon that was identified in previous research [43,44]. The quality of reviewed interventions is variable and the lack of risk management and appropriate adherence to treatment guidelines make it questionable as to whether these products can be recommended to patients suffering from depression without professional oversight. However, many developers are aware of this issue and most web-based interventions do not claim to be a sole therapy tool, but rather a supplement to professional treatment. One reason for this is that high-quality web-based interventions require a wide range of psychotherapeutic, financial, legal, and technological support that cannot always be provided by the developers.

Given the rising demand for web-based treatment options, further research should aim to find principles for the development and evaluation of therapeutically sound web-based interventions for depression, to make is easier for both clinicians and patients to find a suitable product.

### Limitations

This is the first study that investigated the quality of unguided web-based interventions for depression at the level of individual treatment components based on established treatment guidelines. However, some limitations should be regarded when interpreting our findings.

Although we used a standardized questionnaire and trained psychologists to achieve systematic and thorough evaluations, the questionnaire in this study has not yet been externally validated and all ratings are subjective to a certain degree. Further, some beneficial interventions might not be included in this questionnaire. For example, some web-based interventions offered interventions based on problem-solving therapy or systemic therapy, which we did not examine in this study. We consider this questionnaire to be an important first step, but there is room for improvement in the future. A future direction is the external verification of this questionnaire. Additionally, future research should aim to examine a wider range of interventions beyond those based on CBT. As we only used three psychologists to rate the web-based intervention in this study, future research should aim to include more raters with different areas of expertise.

Another limitation is that our raters had no depression diagnosis. Therefore, our raters were not the selected target group of the examined interventions and no conclusion on the effectiveness of web-based interventions for patients can be made. Although the criteria used in this study can be an indicator of high-quality interventions, they do not necessarily have a causal relation to symptom reduction or quality of life improvement, and they cannot be compared to randomized controlled trials involving patients. However, since the aim of this study was not to evaluate effectiveness or symptom reduction, but rather to evaluate the quality of interventions based on their adherence to psychotherapeutic guidelines, we considered the use of expert ratings to be suitable for the purpose of our study. Nevertheless, future research could compare the ratings of patients with the ratings of experts to determine if end users hold different opinions toward the quality of an intervention.

A further limitation of this study is that web-based interventions that required the user to get in touch with a counselor or psychotherapist were not included. Therefore, we can make no claim about how elaborated these interventions are. Additionally, we only evaluated interventions that were available in English or German, as these were the only languages that are fluently spoken by the researchers.

Finally, as we searched for web-based interventions in January 2018 and all our evaluations were performed in 2018, some interventions might no longer be available or may have changed through updates in the meantime.

As a side note, we did not evaluate web-based interventions that used sensor data (eg, through a smartwatch), because we could only identify one such app, which was not available in Germany. As the usage of sensor data is rising in research and practice and promises many advantages to patients, practitioners, and researchers, future research should aim to develop and evaluate web-based interventions that include sensor data.

### Conclusion

Many unguided web-based interventions for depression claim to deliver therapeutic content of high quality, but there is high variability in their adherence to established treatment guidelines and manuals. Although many interventions from manuals are included in web-based interventions and developers offer a wide range of treatment components, the necessary level of therapeutic depth is seldom reached. Mental health professionals and developers should work together to implement current treatment guidelines in their interventions to close this gap, especially concerning the lack of risk management that we identified in our study. This could improve user experience and prevent adverse side effects such as users feeling overwhelmed, frustrated, or leaving the intervention altogether. When developers implement quality criteria of established treatment guidelines in their products, web-based interventions can be a valuable tool to supplement professional treatment. The use of web-based interventions in the treatment of depression enables patients and professionals to gather important information from the patients’ everyday lives and to save financial and time resources because treatment components can be completed at home. There is a need for further research targeting general principles for the development and evaluation of therapeutically sound web-based interventions for depression, which includes treatment interventions beyond CBT.

## Appendix

Multimedia Appendix 1 English version of the questionnaire used in this study.

Multimedia Appendix 2 Web-based interventions tested in this study.

Multimedia Appendix 3 Treatment components that could explicitly be identified for each web-based intervention.

## Abbreviations

CBT: cognitive behavioral therapy
ICC: intraclass correlation coefficient
MARS: Mobile App Rating Scale
