# Supplementary material for: Adherence to Established Treatment Guidelines Among Unguided Digital Interventions for Depression: Quality Evaluation of 28 Web-Based Programs and Mobile Apps
Source: J Med Internet Res. 2020 Jul 13;22(7):e16136. doi: 10.2196/16136 (PMC7385636; doi:10.2196/16136)
Supplement: Multimedia Appendix 1 [file jmir_v22i7e16136_app1.docx]

**Questionnaire for the Evaluation of Web-Based Interventions**

**Program:**

**Evaluator:**

**Testing period (date from - to):**

**Position of the program in the supply chain (Evaluator opinion, multiple choices possible):
 🞎 Prevention 🞎 Bridging 🞎 Treatment 🞎 After-care**

**Self-declaration of the program:
🞎 Prevention 🞎 Bridging 🞎 Treatment 🞎 After-care**

**These interventions were tested:**

**These interventions were not tested:**

**Number of interventions following therapeutic schools: Registered as a medical product: 🞎 Yes
CBT: Psychodynamic: 🞎 No
Systemic: Cross-Schools:
Other:**

| **General evaluation of the intervention**  **Name of the intervention:** | **Strongly disagree** | |  |  | **Strongly agree** |
| --- | --- | --- | --- | --- | --- |
|  | **1** | **2** | **3** | **4** | **5** |
| 1. The therapeutic background for the intervention is presented. | O | O | O | O | O |
| 1. The instructions can easily be understood. | O | O | O | O | O |
| 1. The user can personalize the intervention. | O | O | O | O | O |
| 1. How can the intervention be personalized (keywords)?   ________________________________________________________________________ | | | | | |
| 1. The user can set personal goals. | O | O | O | O | O |
| 1. The user can incorporate individual resources (e.g., personal strengths) into the intervention. | O | O | O | O | O |
| 1. Possible difficulties regarding the intervention are addressed (e.g., lack of perseverance). | O | O | O | O | O |
| 1. The intervention stimulates the recognition of relationship patterns. | O | O | O | O | O |
| 1. The intervention stimulates the recognition of patterns of experiences. | O | O | O | O | O |
| 1. The intervention stimulates the recognition of patterns of behavior. | O | O | O | O | O |
| 1. The (digital) implementation of the intervention is adequate. | O | O | O | O | O |
| 1. The intervention is therapeutically meaningful. | O | O | O | O | O |
| 1. The program regularly reminds the user to perform exercises of the intervention. | O | Yes |  | O | No |
| 1. The user receives positive feedback after completion of the intervention. | O | Yes |  | O | No |
| 1. The intervention can be aborted. | O | Yes |  | O | No |
| 1. The intervention can be skipped. | O | Yes |  | O | No |
| 1. The user can rate the intervention (after completion). | O | Yes |  | O | No |
| 1. The following risks could occur in this intervention:   ________________________________________________________________________ |  |  |  |  |  |

| Behavioral Activation | | | | | | | | | | | |
| --- | --- | --- | --- | --- | --- | --- | --- | --- | --- | --- | --- |
| 1. The program offers suggestions for activities (check all that apply): | | | | | |  |  | |  |  |  |
| 🞎 | activating activities  (e.g., sports) | 🞎 | social activities  (e.g., meeting friends) | 🞎 | relaxing activities  (e.g., taking a bath) |  | **Strongly disagree** | |  |  | **Strongly agree** |
|  |  |  |  |  |  |  | **1** | **2** | **3** | **4** | **5** |
| 1. The suggested activities are easy to realize (in terms of expenses, effort). | | | | | | | O | O | O | O | O |
| 1. The suggested activities are pleasant. | | | | | | | O | O | O | O | O |
| 1. Complex activities are divided into achievable intermediate steps. | | | | | | | O | O | O | O | O |
| 1. Obstacles and difficulties for the realization of suggested activities are explained. | | | | | | | O | O | O | O | O |
| 1. The user can add own activities. | | | | | | | O | Yes |  | O | No |
| 1. The user can schedule future activities. | | | | | | | O | Yes |  | O | No |
| 1. The activities are automatically entered into the calendar of the mobile device. | | | | | | | O | Yes |  | O | No |
| 1. The program asks if a planned activity has been completed. | | | | | | | O | Yes |  | O | No |
| 1. The user can rate an activity after completion. | | | | | | | O | Yes |  | O | No |
| 1. The user receives reminders for planned activities | | | | | | | O | Yes |  | O | No |
| 1. After completion of an activity it is suggested to enter changes in the mood. | | | | | | | O | Yes |  | O | No |
| 1. The program sends suggestions (push notifications) for activities. | | | | | | | O | Yes |  | O | No |
| 1. The level of difficulty of activities is shown. | | | | | | | O | Yes |  | O | No |
| 1. The program reminds the user of positive activities in the past. | | | | | | | O | Yes |  | O | No |
| 1. How many activities are suggested? _____________ | | | | | | |  |  |  |  |  |
| 1. Comments:   ________________________________________________________________________ | | | | | | |  |  |  |  |  |

| **General evaluation of the intervention**  **Name of the intervention:** | **Strongly disagree** | |  |  | **Strongly agree** |
| --- | --- | --- | --- | --- | --- |
|  | **1** | **2** | **3** | **4** | **5** |
| 1. The therapeutic background for the intervention is presented. | O | O | O | O | O |
| 1. The instructions can easily be understood. | O | O | O | O | O |
| 1. The user can personalize the intervention. | O | O | O | O | O |
| 1. How can the intervention be personalized (keywords)?   ________________________________________________________________________ | | | | | |
| 1. The user can set personal goals. | O | O | O | O | O |
| 1. The user can incorporate individual resources (e.g., personal strengths) into the intervention. | O | O | O | O | O |
| 1. Possible difficulties regarding the intervention are addressed (e.g., lack of perseverance). | O | O | O | O | O |
| 1. The intervention stimulates the recognition of relationship patterns. | O | O | O | O | O |
| 1. The intervention stimulates the recognition of patterns of experiences. | O | O | O | O | O |
| 1. The intervention stimulates the recognition of patterns of behavior. | O | O | O | O | O |
| 1. The (digital) implementation of the intervention is adequate. | O | O | O | O | O |
| 1. The intervention is therapeutically meaningful. | O | O | O | O | O |
| 1. The program regularly reminds the user to perform exercises of the intervention. | O | Yes |  | O | No |
| 1. The user receives positive feedback after completion of the intervention. | O | Yes |  | O | No |
| 1. The intervention can be aborted. | O | Yes |  | O | No |
| 1. The intervention can be skipped. | O | Yes |  | O | No |
| 1. The user can rate the intervention (after completion). | O | Yes |  | O | No |
| 1. The following risks could occur in this intervention:   ________________________________________________________________________ |  |  |  |  |  |

| Cognitive Restructuring | | | | | | **Strongly disagree** | |  |  | **Strongly agree** |
| --- | --- | --- | --- | --- | --- | --- | --- | --- | --- | --- |
|  |  |  |  |  |  | **1** | **2** | **3** | **4** | **5** |
| 1. The principle of automatic thoughts is explained clearly. | | | | | | O | O | O | O | O |
| 1. The principle of the cognitive triad (negative views about oneself – negative views about the world – negative views about the future) is explained clearly. | | | | | | O | O | O | O | O |
| 1. The program presents typical negative or stressful thoughts. | | | | | | O | Yes |  | O | No |
| 1. The program encourages the user to write down individual negative or stressful thoughts. | | | | | | O | Yes |  | O | No |
| 1. The program suggests alternative thoughts for negative thoughts. | | | | | | O | Yes |  | O | No |
| 1. The user can add individual alternative thoughts for negative thoughts. | | | | | | O | Yes |  | O | No |
| 1. The user can evaluate thoughts in regard to their credibility. | | | | | | O | Yes |  | O | No |
| 1. The program offers the option to create a daily record of negative thoughts. | | | | | | O | Yes |  | O | No |
| 1. The daily protocol of negative thoughts includes (check all that apply): | | | | | |  |  |  |  |  |
| 🞎 the situation (current events) | | 🞎 the feelings | | 🞎 the intensity of feelings (0-100) | | | | | | |
| 🞎 the negative thoughts | | 🞎 alternative positive thoughts | | 🞎 the results | | | | | | |
| 1. Typical cognitive distortions are explained (check all that apply ): | | | | | |  |  |  |  |  |
| 🞎 | Should Statements | 🞎 | Focus on the Negative | 🞎 | Disqualifying the Positive | | | | | |
| 🞎 | Personalization | 🞎 | Labeling and Mislabeling | 🞎 | Catastrophizing | | | | | |
| 🞎 | Magnification and Minimization | 🞎 | Emotional Reasoning | 🞎 | Jumping to Conclusions | | | | | |
| 🞎 | Polarized Thinking (“Black and White” Thinking) | 🞎 | Overgeneralization |  |  | | | | | |
| 1. Comments:   ________________________________________________________________________ | | | | | |  |  |  |  |  |

| Psychoeducation | **Strongly disagree** | |  |  | **Strongly agree** |
| --- | --- | --- | --- | --- | --- |
|  | **1** | **2** | **3** | **4** | **5** |
| 1. The symptoms of depression are explained clearly. | O | O | O | O | O |
| 1. The program offers an explanatory model for the development of depression. | O | O | O | O | O |
| 1. The user is supported in the development of an individual explanatory model (e.g., select which factors apply in the individual case). | O | O | O | O | O |
| 1. The program appropriately conveys that depressions are well treatable. | O | O | O | O | O |
| 1. The program appropriately conveys that there may be fluctuations in the course of treatment. | O | O | O | O | O |
| 1. The program appropriately conveys that relapses can occur. | O | O | O | O | O |
| 1. The user is encouraged that he/she can overcome a depression. | O | O | O | O | O |
| 1. The role of avoidance behavior is explained clearly. | O | O | O | O | O |
| 1. The role of social isolation is explained clearly. | O | O | O | O | O |
| 1. The possible relationship between depression and anxiety is adequately conveyed. | O | O | O | O | O |
| 1. The possibly increased perception of physical symptoms associated with depression is explained clearly. | O | O | O | O | O |
| 1. Possible somatic causes of physical symptoms are adequately addressed. | O | O | O | O | O |
| 1. Comments:   ________________________________________________________________________ |  |  |  |  |  |

| **General evaluation of the intervention**  **Name of the intervention:** | **Strongly disagree** | |  |  | **Strongly agree** |
| --- | --- | --- | --- | --- | --- |
|  | **1** | **2** | **3** | **4** | **5** |
| 1. The therapeutic background for the intervention is presented. | O | O | O | O | O |
| 1. The instruction is clearly understandable. | O | O | O | O | O |
| 1. The user can personalize the intervention. | O | O | O | O | O |
| 1. How can the intervention be personalized (keywords)?   ________________________________________________________________________ | | | | | |
| 1. The user can set personal goals. | O | O | O | O | O |
| 1. The user can incorporate individual resources (e.g., personal strengths) into the intervention. | O | O | O | O | O |
| 1. Possible difficulties regarding the intervention are addressed (e.g., lack of perseverance). | O | O | O | O | O |
| 1. The intervention stimulates the recognition of relationship patterns. | O | O | O | O | O |
| 1. The intervention stimulates the recognition of patterns of experiences. | O | O | O | O | O |
| 1. The intervention stimulates the recognition of patterns of behavior. | O | O | O | O | O |
| 1. The (digital) implementation of the intervention is adequate. | O | O | O | O | O |
| 1. The intervention is therapeutically meaningful. | O | O | O | O | O |
| 1. The program regularly reminds the user to perform exercises of the intervention. | O | Yes |  | O | No |
| 1. The user receives positive feedback after completion of the intervention. | O | Yes |  | O | No |
| 1. The intervention can be aborted. | O | Yes |  | O | No |
| 1. The intervention can be skipped. | O | Yes |  | O | No |
| 1. The user can rate the intervention (after completion). | O | Yes |  | O | No |
| 1. The following risks could occur in this intervention:   ________________________________________________________________________ |  |  |  |  |  |

| **General evaluation of the intervention**  **Name of the intervention:** | **Strongly disagree** | |  |  | **Strongly agree** |
| --- | --- | --- | --- | --- | --- |
|  | **1** | **2** | **3** | **4** | **5** |
| 1. The therapeutic background for the intervention is presented. | O | O | O | O | O |
| 1. The instructions can easily be understood. | O | O | O | O | O |
| 1. The user can personalize the intervention. | O | O | O | O | O |
| 1. How can the intervention be personalized (keywords)?   ________________________________________________________________________ | | | | | |
| 1. The user can set personal goals. | O | O | O | O | O |
| 1. The user can incorporate individual resources (e.g., personal strengths) into the intervention. | O | O | O | O | O |
| 1. Possible difficulties regarding the intervention are addressed (e.g., lack of perseverance). | O | O | O | O | O |
| 1. The intervention stimulates the recognition of relationship patterns. | O | O | O | O | O |
| 1. The intervention stimulates the recognition of patterns of experiences. | O | O | O | O | O |
| 1. The intervention stimulates the recognition of patterns of behavior. | O | O | O | O | O |
| 1. The (digital) implementation of the intervention is adequate. | O | O | O | O | O |
| 1. The intervention is therapeutically meaningful. | O | O | O | O | O |
| 1. The program regularly reminds the user to perform exercises of the intervention. | O | Yes |  | O | No |
| 1. The user receives positive feedback after completion of the intervention. | O | Yes |  | O | No |
| 1. The intervention can be aborted. | O | Yes |  | O | No |
| 1. The intervention can be skipped. | O | Yes |  | O | No |
| 1. The user can rate the intervention (after completion). | O | Yes |  | O | No |
| 1. The following risks could occur in this intervention:   ________________________________________________________________________ |  |  |  |  |  |

| Mood Tracking | **Strongly disagree** | |  |  | **Strongly agree** |
| --- | --- | --- | --- | --- | --- |
|  | **1** | **2** | **3** | **4** | **5** |
| 1. The user is asked about his/her mood. | O | Yes |  | O | No |
| 1. The query of the mood is adequately visualized (e.g., mood barometer). | O | O | O | O | O |
| 1. The query of the mood is appropriately scaled (at least 5 options). | O | Yes |  | O | No |
| 1. The query takes appropriate account of common diagnostic criteria for depression. | O | O | O | O | O |
| 1. The program visualizes the mood progression (e.g., as a curve). | O | Yes |  | O | No |
| 1. The user is asked about anxiety symptoms. | O | Yes |  | O | No |
| 1. The user is asked about physical ailments. | O | Yes |  | O | No |
| 1. The program advises to consult a doctor for physical ailments. | O | Yes |  | O | No |
| 1. When the mood progression is visualized, small periods of time (the last hours/the present day) can be selected | O | Yes |  | O | No |
| 1. When the mood progression is visualized, large periods of time (more than a week) can be selected | O | Yes |  | O | No |
| 1. The program suggests possible relations between the mood and current events. | O | Yes |  | O | No |
| 1. The program highlights mood improvements. | O | Yes |  | O | No |
| 1. The importance of small periods of time to detect triggers in the mood progression is explained clearly. | O | O | O | O | O |
| 1. The importance of large periods of time to detect a mood trend is explained clearly. | O | O | O | O | O |
| 1. How often is the mood queried?   at least every 2 hrs. every 2-4 hrs. every 4-6 hrs. less than every 6 h.  O O O O |  |  |  |  |  |
| 1. Comments:   ________________________________________________________________________ |  |  |  |  |  |

| **General evaluation of the intervention**  **Name of the intervention:** | **Strongly disagree** | |  |  | **Strongly agree** |
| --- | --- | --- | --- | --- | --- |
|  | **1** | **2** | **3** | **4** | **5** |
| 1. The therapeutic background for the intervention is presented. | O | O | O | O | O |
| 1. The instructions can easily be understood. | O | O | O | O | O |
| 1. The user can personalize the intervention. | O | O | O | O | O |
| 1. How can the intervention be personalized (keywords)?   ________________________________________________________________________ | | | | | |
| 1. The user can set personal goals. | O | O | O | O | O |
| 1. The user can incorporate individual resources (e.g., personal strengths) into the intervention. | O | O | O | O | O |
| 1. Possible difficulties regarding the intervention are addressed (e.g., lack of perseverance). | O | O | O | O | O |
| 1. The intervention stimulates the recognition of relationship patterns. | O | O | O | O | O |
| 1. The intervention stimulates the recognition of patterns of experiences. | O | O | O | O | O |
| 1. The intervention stimulates the recognition of patterns of behavior. | O | O | O | O | O |
| 1. The (digital) implementation of the intervention is adequate. | O | O | O | O | O |
| 1. The intervention is therapeutically meaningful. | O | O | O | O | O |
| 1. The program regularly reminds the user to perform exercises of the intervention. | O | Yes |  | O | No |
| 1. The user receives positive feedback after completion of the intervention. | O | Yes |  | O | No |
| 1. The intervention can be aborted. | O | Yes |  | O | No |
| 1. The intervention can be skipped. | O | Yes |  | O | No |
| 1. The user can rate the intervention (after completion). | O | Yes |  | O | No |
| 1. The following risks could occur in this intervention:   ________________________________________________________________________ |  |  |  |  |  |

| Journal Keeping | **Strongly disagree** | |  |  | **Strongly agree** |
| --- | --- | --- | --- | --- | --- |
|  | **1** | **2** | **3** | **4** | **5** |
| 1. The program explains clearly which components journal entries might contain (e.g., activities, events, social contacts, feelings etc.). | O | O | O | O | O |
| 1. The program explains clearly that it can be helpful to note the positive aspects of the day. | O | O | O | O | O |
| 1. Journal entries are taken up in the progression of the program. | O | Yes |  | O | No |
| 1. The user receives feedback on the content of his/her journal entries. | O | Yes |  | O | No |
| 1. The program reminds the user (push notification) to make journal entries on a regular basis. | O | Yes |  | O | No |
| 1. The program proposes phrasings for particular aspects of the journal. | O | Yes |  | O | No |
| 1. Comments:   ________________________________________________________________________ |  |  |  |  |  |

| **General evaluation of the intervention**  **Name of the intervention:** | **Strongly disagree** | |  |  | **Strongly agree** |
| --- | --- | --- | --- | --- | --- |
|  | **1** | **2** | **3** | **4** | **5** |
| 1. The therapeutic background for the intervention is presented. | O | O | O | O | O |
| 1. The instructions can easily be understood. | O | O | O | O | O |
| 1. The user can personalize the intervention. | O | O | O | O | O |
| 1. How can the intervention be personalized (keywords)?   ________________________________________________________________________ | | | | | |
| 1. The user can set personal goals. | O | O | O | O | O |
| 1. The user can incorporate individual resources (e.g., personal strengths) into the intervention. | O | O | O | O | O |
| 1. Possible difficulties regarding the intervention are addressed (e.g., lack of perseverance). | O | O | O | O | O |
| 1. The program regularly reminds the user to perform exercises of the intervention. | O | O | O | O | O |
| 1. The intervention stimulates the recognition of relationship patterns. | O | O | O | O | O |
| 1. The intervention stimulates the recognition of patterns of experiences. | O | O | O | O | O |
| 1. The intervention stimulates the recognition of patterns of behavior. | O | O | O | O | O |
| 1. The (digital) implementation of the intervention is adequate. | O | O | O | O | O |
| 1. The intervention is therapeutically meaningful. | O | O | O | O | O |
| 1. The user receives positive feedback after completion of the intervention. | O | O | O | O | O |
| 1. The intervention can be aborted. | O | Yes |  | O | No |
| 1. The intervention can be skipped. | O | Yes |  | O | No |
| 1. The user can rate the intervention (after completion). | O | Yes |  | O | No |
| 1. The following risks could occur in this intervention:   ________________________________________________________________________ |  |  |  |  |  |

| Relaxation | **Strongly disagree** | |  |  | **Strongly agree** |
| --- | --- | --- | --- | --- | --- |
|  | **1** | **2** | **3** | **4** | **5** |
| 1. The concept of mindfulness is explained clearly. | O | O | O | O | O |
| 1. The program suggests accepting perceptions without judging them (non-judging attitude). | O | Yes |  | O | No |
| 1. The program suggests mindfulness exercises for the daily routine (e.g., mindful tooth brushing). | O | Yes |  | O | No |
| 1. The program suggests distancing oneself from thoughts (e.g., imagining oneself to be spatially distant, viewing oneself as an external observer). | O | Yes |  | O | No |
| 1. The program offers audios/videos with mindfulness exercises. | O | Yes |  | O | No |
| 1. The user can create a schedule with regular mindfulness exercises. | O | Yes |  | O | No |
| 1. The user can add personal stressors. | O | Yes |  | O | No |
| 1. Typical stressors are explained clearly. | O | O | O | O | O |
| 1. Possible risks in performing mindfulness exercises (e.g., the occurrence of unpleasant feelings) are explained clearly. | O | O | O | O | O |
| 1. Possible difficulties in performing mindfulness exercises (e.g., boredom, falling asleep) are explained clearly. | O | O | O | O | O |
| 1. The program offers other relaxation techniques (check all that apply):   🞎 PMR 🞎 Autogenic Training 🞎 Meditation 🞎 Guided imagery journeys 🞎 Imagination exercises 🞎 Hypnosis | | | | | |
| 1. Comments:   ________________________________________________________________________ |  |  |  |  |  |

| **General evaluation of the intervention**  **Name of the intervention:** | **Strongly disagree** | |  |  | **Strongly agree** |
| --- | --- | --- | --- | --- | --- |
|  | **1** | **2** | **3** | **4** | **5** |
| 1. The therapeutic background for the intervention is presented. | O | O | O | O | O |
| 1. The instructions can easily be understood. | O | O | O | O | O |
| 1. The user can personalize the intervention. | O | O | O | O | O |
| 1. How can the intervention be personalized (keywords)?   ________________________________________________________________________ | | | | | |
| 1. The user can set personal goals. | O | O | O | O | O |
| 1. The user can incorporate individual resources (e.g., personal strengths) into the intervention. | O | O | O | O | O |
| 1. Possible difficulties regarding the intervention are addressed (e.g., lack of perseverance). | O | O | O | O | O |
| 1. The intervention stimulates the recognition of relationship patterns. | O | O | O | O | O |
| 1. The intervention stimulates the recognition of patterns of experiences. | O | O | O | O | O |
| 1. The intervention stimulates the recognition of patterns of behavior. | O | O | O | O | O |
| 1. The (digital) implementation of the intervention is adequate. | O | O | O | O | O |
| 1. The intervention is therapeutically meaningful. | O | O | O | O | O |
| 1. The program regularly reminds the user to perform exercises of the intervention. | O | Yes |  | O | No |
| 1. The user receives positive feedback after completion of the intervention. | O | Yes |  | O | No |
| 1. The intervention can be aborted. | O | Yes |  | O | No |
| 1. The intervention can be skipped. | O | Yes |  | O | No |
| 1. The user can rate the intervention (after completion). | O | Yes |  | O | No |
| 1. The following risks could occur in this intervention:   ________________________________________________________________________ |  |  |  |  |  |

| Social Skills Training | **Strongly disagree** | |  |  | **Strongly agree** |
| --- | --- | --- | --- | --- | --- |
|  | **1** | **2** | **3** | **4** | **5** |
| 1. The program involves relatives of the user (e.g., in exercises). | O | O | O | O | O |
| 1. The program involves (fictional) people in the exercises. | O | Yes |  | O | No |
| 1. The (fictional) people are appropriately represented (concerning their attributes). | O | O | O | O | O |
| 1. The program encourages a change of perspective. | O | O | O | O | O |
| 1. The program offers standard situation of social interaction (pre-formulated scenes). | O | Yes |  | O | No |
| 1. Non-verbal components of social interactions (e.g., keeping eye contact) are explained clearly. | O | O | O | O | O |
| 1. Verbal components of social interactions (e.g., voice modulation) are explained clearly. | O | O | O | O | O |
| 1. The program encourages the user to perform exercises of social interaction (e.g., paying somebody a compliment). | O | Yes |  | O | No |
| 1. The perception of social cues is adequately addressed. | O | O | O | O | O |
| 1. The establishing and maintaining of social contacts is adequately addressed. | O | O | O | O | O |
| 1. The adequate assertion of one’s own wishes in social situations is adequately addressed (e.g., the ability to say ‘No’). | O | O | O | O | O |
| 1. Behavior in conflict situations is adequately addressed. | O | O | O | O | O |
| 1. The principle of social roles and possible role conflicts is explained understandingly. | O | O | O | O | O |
| 1. Comments:   ________________________________________________________________________ |  |  |  |  |  |
|  |  |  |  |  |  |

| Crisis Management | **Strongly disagree** | |  |  | **Strongly agree** |
| --- | --- | --- | --- | --- | --- |
|  | **1** | **2** | **3** | **4** | **5** |
| 1. The therapeutic background for the intervention is presented. | O | O | O | O | O |
| 1. The instructions can easily be understood. | O | O | O | O | O |
| 1. The user can incorporate individual resources (e.g., personal strengths) into the intervention. | O | O | O | O | O |
| 1. The (digital) implementation of the intervention is adequate. | O | O | O | O | O |
| 1. The intervention is therapeutically meaningful. | O | O | O | O | O |
| 1. In order the use the program, the user must provide an emergency contact. | O | Yes |  | O | No |
| 1. The program provides emergency contacts (contact details that can be viewed at any time). | O | Yes |  | O | No |
| 1. The user can create an emergency plan with individual measures. | O | Yes |  | O | No |
| 1. Possible difficulties to the implementation of the emergency plan (e.g., emergency contact is not available) are addressed adequately. | O | Yes |  | O | No |
| 1. The program suggests contacting the emergency contact when the mood (in the mood tracking) has dropped immensely. | O | Yes |  | O | No |
| 1. The program suggests resorting to the emergency plan when the mood (in the mood tracking) has dropped immensely. | O | Yes |  | O | No |
| 1. The program offers contact to support groups. | O | Yes |  | O | No |
| 1. The user can create an individual list of warning signs in case of a relapse. | O | Yes |  | O | No |
| 1. Comments or possible risks:   ________________________________________________________________________ |  |  |  |  |  |

| Resource Activation | **Strongly disagree** | |  |  | **Strongly agree** |
| --- | --- | --- | --- | --- | --- |
|  | **1** | **2** | **3** | **4** | **5** |
| 1. The user is encouraged to identify his/her own resources. | O | O | O | O | O |
| 1. The program provides adequate assistance in identifying individual resources (e.g., thinking about past successes). | O | O | O | O | O |
| 1. The user is encouraged to reflect resources in their context (e.g., origin story, typical situations, promoting or inhibiting factors). | O | O | O | O | O |
| 1. Resources are taken up as the program progresses. | O | Yes |  | O | No |
| 1. Comments:   ________________________________________________________________________ |  |  |  |  |  |

## Notes

(e.g., how often was the program used in a day; how long did one session take; is there a schedule?)

**First Measurement:**

**Key symptoms**:
Depressed Mood
Fatigue or low energy
Loss of interest or pleasure

Mild to moderate severity of symptoms (about 20%-45% of symptoms fulfilled)

**Second Measurement:**

**Increased severity regarding:**Depressed Mood
Fatigue or low energy
Loss of interest or pleasure
Suicidal thoughts
Feelings of worthlessness / Feelings of guilt
Decrease or increase of appetite / Sleep disturbances

**Third Measurement:**

Improvement of symptoms
Only mild severity of symptoms, some symptoms are completely remitted

**Reminder: Symptom severity**

**21 Symptoms:**

1. Depressed mood

2. Pessimism

3. Low self-esteem

4. Self-dissatisfaction

5. Feelings of guilt

6. Feelings of being punished

7. Self-rejection

8. Self-reproaches

9. Thoughts of suicide

10. Crying

11. Irritability

12. Social isolation

13. Indecisiveness

14. Feelings of worthlessness

15. Loss of energy

16. Sleep disturbances

17. Fatigue

18. Decreased or increased appetite

19. Agitation

20. Diminished ability to concentrate

21. Loss of libido
