# Supplementary material for: Adherence to Established Treatment Guidelines Among Unguided Digital Interventions for Depression: Quality Evaluation of 28 Web-Based Programs and Mobile Apps
Source: J Med Internet Res. 2020 Jul 13;22(7):e16136. doi: 10.2196/16136 (PMC7385636; doi:10.2196/16136)
Supplement: Multimedia Appendix 2 [file jmir_v22i7e16136_app2.pdf]

Reference #	Program Name	Type of Program (Smartphone Application or Desktop Program)	Provider	Link
1	15minutes4me	DP	Coachteam BVBA	https://www.15minutes4me.com/
2	Arya Companion	App	Arya mHealth UG	https://www.aryaapp.co/
3	Beating the Blues	DP	365 Health and Wellbeing Ltd	http://www.beatingtheblues.co.uk/
4	CBT Worry Thought Journal	App	Adventuroo Apps	https://play.google.com/store/apps/details?id=com.adventuroo.happiness&hl=en
5	Cognitive Behavioral Therapy: Depression & Anxiety	App	NKDigital	https://play.google.com/store/apps/details?id=com.dmbteam.cbt
6	Depression CBT Selfhelp Guide	App	Excel at Life	https://play.google.com/store/apps/details?id=com.excelatlife.depression
7	Depressiv? Was hilft?	App	Psychotherapeutische Praxis Martin Brentrup	https://play.google.com/store/apps/details?id=com.depressiv.pro
8	Ecouch	DP	eHub Health Pty Ltd	https://ecouch.anu.edu.au/
9	Emotion	App	Les Laboratiores Servier	https://play.google.com/store/apps/details?id=com.servier.emotion_plus
10	Evolution Health	DP	Evolution Health Systems	https://evolutionhealth.care/
11	Hausmed Coaching Depression (Raus aus dem Tief)	DP	Dr. Becker eHealth GmbH	https://www.hausmed.de/hausmedcoach/depression/
12	Learn to Live	DP	Learn to Live, Inc.	https://www.learntolive.com/
13	Mood Sentry	App	Todd M. Williams	https://play.google.com/store/apps/details?id=com.MoodApps.MoodSentry
14	MoodSpace	App	Boundless	https://play.google.com/store/apps/details?id=boundless.moodgym
15	MoodTools - Depression Aid	App	MoodTools	https://play.google.com/store/apps/details?id=com.moodtools.moodtools
16	Mood Triggers: Anxiety Depression Insomnia Tracker	App	Nicholas C. Jacobson	http://www.nicholasjacobson.com/project/mood-triggers/
17	Moodfit - Stress & Anxiety	App	Roble Ridge Software LLC	https://play.google.com/store/apps/details?id=com.robleridge.Moodfit
18	Moodgym	DP	ehub Health Pty Ltd	https://moodgym.de/
19	Moodpath: Depression & Burnout Test	App	Moodpath	https://play.google.com/store/apps/details?id=de.moodpath.android
20	My Depression Hack	App	Sada Yogini	https://play.google.com/store/apps/details?id=com.goodbarber.cbt123
21	novego	DP	IVPNetworks GmbH	https://www.novego.de/
22	Pacifica - Stress & Anxiety	App	Pacifica Labs Inc.	https://play.google.com/store/apps/details?id=com.pacificalabs.pacifica
23	Psychonline	DP	Neuropsychiatrisches Zentrum Hamburg-	https://www.psychonline.de/
24	Psychosomat Depression Burnout	App	Christian Brecht	https://play.google.com/store/apps/details?id=cbsoft.psychosomat
25	Selfapy	DP	Selfapy GmbH	https://www.selfapy.de/
26	Stimmungstagebuch	App	Börm Bruckmeier Verlag GmbH	https://play.google.com/store/apps/details?id=com.bbi.stimmungstagebuch
27	The Journal	DP	Health Promotion Agency	https://depression.org.nz/get-better/the-journal/
28	What's up?	App	Jackson Tempra	https://play.google.com/store/apps/details?id=com.jacksontempra.apps.whatsup

DP: desktop program
App: smartphone application
