# Supplementary material for: Adherence to Established Treatment Guidelines Among Unguided Digital Interventions for Depression: Quality Evaluation of 28 Web-Based Programs and Mobile Apps
Source: J Med Internet Res. 2020 Jul 13;22(7):e16136. doi: 10.2196/16136 (PMC7385636; doi:10.2196/16136)
Supplement: Multimedia Appendix 3 [file jmir_v22i7e16136_app3.pdf]

Reference #	Program Name	Type of Program	Behavioral activation	Cognitive Restructuring	Psycho-education	Mood Tracking	Journal Keeping	Relaxation	Social Skills Training	Crisis Management
1	15minutes4me	DP			✔	✔				
2	Arya Companion	App	✔		✔	✔				✔
3	Beating the Blues	DP	✔	✔	✔	✔	✔			✔
4	CBT Worry Thought Journal	App		✔		✔				
5	Therapy: Depression & Anxiety	App	✔	✔	✔				✔	
6	Guide	App	✔	✔	✔	✔	✔	✔		✔
7	Depressiv? Was hilft?	App	✔	✔	✔	✔		✔	✔	✔
8	Ecouch	DP	✔	✔	✔	✔		✔	✔	✔
9	Emotion	App			✔	✔		✔		
10	Evolution Health	DP	✔	✔	✔	✔	✔			
11	Depression (Raus aus dem Tief)	DP	✔	✔	✔	✔	✔	✔	✔	✔
12	Learn to Live	DP	✔	✔	✔	✔		✔	✔	✔
13	Mood Sentry	App		✔	✔		✔			
14	MoodSpace	App		✔			✔	✔		
15	MoodTools - Depression Aid	App	✔	✔	✔	✔	✔	✔		✔
16	Mood Triggers: Anxiety Depression Insomnia Tracker	App				✔				
17	Moodfit - Stress & Anxiety	App	✔	✔		✔		✔		✔
18	Moodgym	DP	✔	✔	✔	✔		✔	✔	✔
19	Burnout Test	App	✔	✔	✔	✔	✔	✔		✔
20	My Depression Hack	App		✔				✔		✔
21	novego	DP	✔	✔	✔	✔		✔	✔	✔
22	Pacifica - Stress & Anxiety	App	✔	✔	✔	✔	✔	✔		✔
23	Psychonline	DP				✔		✔		✔
24	Burnout	App		✔		✔	✔			✔
25	Selfapy	DP	✔	✔	✔	✔	✔	✔	✔	✔
26	Stimmungstagebuch	App	✔		✔	✔				✔
27	The Journal	DP	✔		✔	✔		✔		✔
28	What's up?	App	✔	✔	✔	✔	✔	✔		✔

" ✔ ": component could explicetly be identified by the raters

DP: desktop program

App: smartphone application
